# C-type lectin receptor Dectin-1 blockade on tumour-associated macrophages improves anti-PD-1 efficacy in gastric cancer

**DOI:** 10.1038/s41416-023-02336-5

**Published:** 2023-07-08

**Authors:** Xin Liu, Kunpeng Lv, Jieti Wang, Chao Lin, Hao Liu, Heng Zhang, He Li, Yun Gu, Ruochen Li, Hongyong He, Jiejie Xu

**Affiliations:** 1 NHC Key Laboratory of Glycoconjugate Research, Department of Biochemistry and Molecular Biology, School of Basic Medical Sciences, Fudan University, Shanghai, China; 2 Department of Endoscopy, Fudan University Shanghai Cancer Center, Shanghai, China; 3 Department of General Surgery, Zhongshan Hospital, Fudan University, Shanghai, China; 4 Department of General Surgery, Shanghai Sixth People’s Hospital, Shanghai Jiao Tong University School of Medicine, Shanghai, China

**Keywords:** Gastric cancer, Monocytes and macrophages, Cancer microenvironment, Immune evasion, Immunosuppression

## Abstract

**Background:**

This study aimed to investigate the expression and clinical significance of Dendritic cell-associated C-type lectin-1 (Dectin-1) in gastric cancer (GC), and to explore the mechanism of Dectin-1 regulating tumour-associated macrophage (TAM)-mediated immune evasion in GC.

**Methods:**

The association of Dectin-1^+^ cells with clinical outcomes was inspected by immunohistochemistry on tumour microarrays. Flow cytometry and RNA sequencing were applied to detect characteristics of T cells, phenotypic and transcriptional features of Dectin-1^+^ TAMs. The effect of Dectin-1 blockade was evaluated using an in vitro intervention experiment based on fresh GC tissues.

**Results:**

High infiltration of intratumoral Dectin-1^+^ cells predicted poor prognosis in GC patients. Dectin-1^+^ cells were mainly composed of TAMs, and the accumulation of Dectin-1^+^ TAMs was associated with T-cell dysfunction. Notably, Dectin-1^+^ TAMs exhibited an immunosuppressive phenotype. Furthermore, blockade of Dectin-1 could reprogramme Dectin-1^+^ TAMs and reactivate anti-tumour effects of T cells, as well as enhanced PD-1 inhibitor-mediated cytotoxicity of CD8^+^ T cells against tumour cells.

**Conclusions:**

Dectin-1 could affect T-cell anti-tumour immune response by regulating the immunosuppressive function of TAMs, leading to poor prognosis and immune evasion in GC patients. Blockade of Dectin-1 can be used alone or in combination with current therapeutic strategies in GC.

## Introduction

Despite a decrease in mortality, gastric cancer (GC) remains the fifth most common cancer type and the fourth leading cause of global cancer-related death [1]. For decades, chemotherapy has been the backbone of the treatment for advanced GC [2]. Molecular targeted agents such as human epidermal growth factor receptor 2 (HER2) and epidermal growth factor receptor (EGFR) monoclonal antibodies have been proven to be effective in certain GC patients; however, options are still limited, and the median overall survival (OS) for advanced GC patients is only about 1 year [3, 4]. With the deepening of the tumour microenvironment, immunotherapy based on reactivation of anti-tumour immune responses has recently yielded considerable progress in several tumours [5]. Nevertheless, clinical trials have suggested that only a fraction of GC patients showed response to treatment, highlighting the importance of developing novel therapeutic approaches [6].

To date, the majority of immunotherapies have focused on reversing T-cell exhaustion to attack tumour cells, including immune checkpoint inhibitors (ICIs) targeting the programmed death receptor-1 (PD-1)/programmed death ligand-1 (PD-L1) axis. In addition to directly suppressing T-cell immune responses mediated by immune checkpoints, accumulating evidence have suggested that tumour cells can also educate myeloid cells to induce immune evasion [7]. Tumour-associated macrophages (TAMs), a major component of myeloid cells within the tumour microenvironment, can affect tumour immunity and influence therapeutic responsiveness [8]. Specifically, TAMs are very receptive to changes in the tumour microenvironment and are highly heterogeneous, which include immunostimulatory and immunosuppressive subsets [9, 10]. Therefore, reprograming immunosuppressive TAMs is an attractive treatment strategy and may be complementary or synergistic in combination with existing treatment regimens [11].

Dendritic cell-associated C-type lectin-1 (Dectin-1), also known as *CLEC7A*, belongs to the C-type lectin receptor (CLR) family and is predominantly expressed in myeloid cells, including macrophages and dendritic cells (DCs) [12]. Dectin-1 has been extensively studied due to its high affinity for β-1,3-glucans present in fungal cell walls and triggers anti-fungal immune responses [13, 14]. However, the function of Dectin-1 in sterile inflammation, especially in tumours, has not been reported until recently [15, 16]. Studies have shown that Dectin-1-activated DCs can induce the anti-tumour effects of Th9 cells and Dectin-1 is essential for natural killer (NK) cell-mediated killing of tumour cells expressing N-glycans [17, 18]. On the contrary, high expression of Dectin-1 is associated with poor prognosis in clear cell renal cell carcinoma and promote pancreatic cancer tumorigenesis by inducing immune tolerance [19, 20]. More importantly, targeting the galectin-9/Dectin-1 axis with an exosome-based dual delivery biosystem can reverse the immunosuppression of M2-like TAMs and enhance anti-tumour immunity, suggesting that Dectin-1 is a valuable target for cancer immunotherapy [21]. Nevertheless, the impact of Dectin-1 on tumour immunity in GC has not been addressed.

Here, we discovered that Dectin-1 was highly expressed in tumour tissues and could be an independent prognostic factor for predicting poor prognosis in GC. Furthermore, we demonstrated that Dectin-1^+^ cells were mainly composed of TAMs in GC, and Dectin-1^+^ TAMs possessed an immunosuppressive phenotype that mediated T-cell dysfunction. We also found that using a Dectin-1-neutralising antibody could reverse the immunosuppressive effects of TAMs, thereby reactivating the anti-tumour immune response of T cells. Importantly, the combination of anti-Dectin-1 antibody anti-PD-1 enhanced anti-tumour activity. These findings suggest that Dectin-1 is expected to become a novel immunotherapy target for GC.

## Materials and methods

### Patients and follow-up

This study included 496 GC patients from Zhongshan Hospital, Fudan University (Shanghai, China), which underwent radical gastrectomy and standard D2 lymphadenectomy between August 2007 and December 2008. Samples including tumour and adjacent peritumor tissues were formalin-fixed, paraffin-embedded (FFPE) and constructed into tissue microarray (TMA) slides. The tumour dot was derived from the tumour centre area, whereas the peritumor dot was acquired from the area ≥2 cm from the tumour margin. Forty-five patients were excluded due to data missing, metastatic diseases or dot loss. Then, the other 451 patients were randomly separated into two independent datasets, the Discovery set (*n* = 200) and the Validation set (*n* = 251). Details about the clinicopathological characteristics of GC patients in these two datasets are shown in Supplementary Table S1. The pathological tumour (pT) stage, pathological node (pN) stage and tumour–node–metastasis (TNM) stage were evaluated based on the 7th edition of the American Joint Committee on Cancer TNM staging system.

The end-point of interest was OS and disease-free survival (DFS). OS was defined as the time interval from the date of resection to death or the last follow-up, and DFS was defined as the time interval from the date of resection to the date of first recurrence or the last follow-up. Patients were followed up until April 2014. Written informed consent was obtained from each patient. This study followed the guidelines of the Declaration of Helsinki and was approved by the Clinical Research Ethics Committee of Zhongshan Hospital, Fudan University (Shanghai, China).

### Gene expression analysis

The mRNA expression data are available from The Cancer Genome Atlas (TCGA) database (https://xena.ucsc.edu/) and Gene Expression Omnibus (GEO) database (https://www.ncbi.nlm.nih.gov/geo/). In addition, this study included 61 GC patients treated with pembrolizumab recruited from the European Nucleotide Archive (ENA: http://www.ebi.ac.uk/ena). However, 16 of them were excluded due to lack of transcriptional data, and the remaining 45 GC patients with drug response information were further analysed, including complete response (CR), partial response (PR), progressive disease (PD), and stable disease (SD).

### Immunohistochemistry and immunofluorescence

For immunohistochemistry (IHC) staining, FFPE TMA slides were deparaffinized, rehydrated in graded alcohol, blocked by endogenous peroxidase blocking solution and subjected to antigen retrieval using 10 mM citrate buffer. TMA slides were then blocked with goat serum for 2 h at 37 °C and incubated overnight with primary antibody: Dectin-1 (ab140039, Abcam) at 4 °C. Subsequently, slides were incubated with a horseradish peroxidase (HRP)-conjugated secondary antibody, followed by stain using 3,3’-diaminobenzidine solution. Finally, slides were counterstained with hematoxylin and mounted with neutral balsam. The number of Dectin-1^+^ cells in each slide was quantified by two independent observers who were blinded to clinicopathological information of GC patients. The interclass correlation between the two pathologists’ evaluation for Dectin-1^+^ cells from the same cohort was 0.954 (95% CI: 0.944–0.961, *P* < 0.001). The mean count of their evaluation was adopted.

For immunofluorescence staining, FFPE TMA slide preparation, antigen retrieval and serum blockade were performed as described above. Then, two staining processes were performed sequentially, including separate incubations with primary antibodies: Dectin-1 (ab140039, Abcam) and CD68 (ab955, Abcam) for 1 h at 37 °C, a HRP-conjugated secondary antibody for 10 min at 37 °C, and the tyramide signal amplification (TSA) buffer with corresponding fluorescent dyes: TSA620 (D110014, WiSee Biotechnology) and TSA520 (D110011, WiSee Biotechnology) for 10 min at 37 °C. Finally, the sections were incubated with DAPI and mounted with anti-fade mounting medium. All stained sections were imaged by Case Viewer 2.3.

### Single-cell suspensions preparation of human GC tissues

A total of 61 tissue samples were obtained from GC patients, who underwent gastrectomy from Zhongshan Hospital, Fudan University (Shanghai, China) from August 2020 to January 2022. Written informed consent was obtained from all patients, and protocols were approved by the Clinical Research Ethics Committee of Zhongshan Hospital. Fresh GC tissues were collected immediately after the samples were resected during the surgery. To prepare single-cell suspensions, tissue samples were manually minced and digested in RPMI1640 medium containing 1 mg/ml Collagenase IV (Sigma Aldrich), 0.1 mg/ml deoxyribonuclease I (DNase I) (Roche) for 2 h at 37 °C on a shaker. GolgiStop Protein Transport Inhibitor (Containing Monensin, BD Bioscience) were added depending on whether intracellular protein detection was performed. Next, the tissue digestion was filtered through a 70-μm cell strainer (BD Falcon) and washed once with Stain Buffer (BD Bioscience). Cells were incubated with RBC Lysing Buffer (BD Bioscience) for 10 min to remove red blood cells (RBCs).

### Flow cytometry

Single-cell suspensions were incubated with Fixable Viability Stain 510 for 15 min to delineate between live and dead cells. Human Fc-receptor-blocking antibody (BD Bioscience) was then added and incubated for 15 min to reduce non-specific binding. Subsequently, cells were surface-stained with corresponding antibodies for 30 min at 4 °C in the dark. To stain for intracellular proteins or transcription factors, surface antigen-labelled cells were fixed and permeabilized by Fixation/Permeabilization Buffer or Transcription Factor Fixation/Permeabilization Buffer (BD Bioscience) and then stained, respectively. After washing with permeabilization buffer, the stained cells were suspended in Stain Buffer at 4 °C and acquired on a BD FACS Celesta flow cytometer. FlowJo Software (Tree Star) was used for data analysis. All flow cytometry (FCM) antibodies are summarised in Supplementary Table S2.

### Fluorescence-activated cell sorting and sample preparation for RNA sequencing

Fluorescence-activated cell sorting (FACS) was performed using mononuclear cells of GC tumour tissues. Briefly, tumour single-cell suspensions were enriched by 40% and 70% Percoll gradient (17089102; GE Life) and RBC Lysing Buffer, and centrifugated to separate mononuclear cells. After PBS washes, the mononuclear cells were stained with CD45, CD14 and Dectin-1. Then, Dectin-1^+^TAMs (CD45^+^CD14^+^Dectin-1^+^) and Dectin-1^−^TAMs (CD45^+^CD14^+^Dectin-1^−^) were sorted using the MoFlo XDP (Beckman Coulter). Finally, sorted cells were vortexed and lysed in lysis buffer and immediately frozen with liquid nitrogen for RNA sequencing (RNA-seq). Antibodies are listed in Supplementary Table S2.

### RNA-seq and analysis

Standard Smart-seq2 protocols were applied for construction of cDNA libraries [22]. Libraries were analysed on Illumina NovaSeq 6000 platform. HISAT2 software was used to align sequencing data with the human transcriptome data [23]. Thereafter, the gene counts data of Dectin-1^+^ TAMs and Dectin-1^−^ TAMs were used for further analysis. To identify the differentially expressed genes (DEGs), the DESeq2 method in Sangerbox Tools (http://vip.sangerbox.com) was used. Heatmaps of gene expression levels were generated using OmicStudio tool (https://www.omicstudio.cn/tool/4). To determine the functional annotation of the differentially expressed gene, Gene Ontology (GO) analysis was performed using DAVID (https://david.ncifcrf.gov/tools.jsp) and the biological process (BP) pathway annotation of genes were represented using Lollipop chart by Sangerbox Tools. Gene set enrichment analysis (GSEA) was also applied for gene functional annotation.

### In vitro intervention assay

Tumour single-cell suspensions were prepared as described above. In this study, tumour single-cell suspensions were divided into two treatment groups: Isotype group (Mouse IgG1 isotype control antibody, clone# T8E5, cat# mabg1-ctrlm, InvivoGen, 10 μg/mL) and Anti-Dectin-1 group (Human Dectin-1 monoclonal antibody, clone# 22H8, cat# mabg-hdect, InvivoGen, 10 μg/mL). Each treatment group was cultured in RPMI1640 medium (Gibco) containing 10% foetal bovine serum (FBS, Gibco) for 12 h at 37 °C. Functionally, the anti-Dectin-1 neutralising antibody blocks Dectin-1-induced cellular activation [24]. After overnight culture, the suspensions were subjected to FCM for TAM and T-cell analysis. To test whether anti-Dectin-1 treatment could augment therapeutic efficacy of PD-1/PD-L1 blockade, Tumour single-cell suspensions were treated with anti-Dectin-1 antibody either alone or in combination with anti-PD-1 antibody (Camrelizumab, 201904001F, Suzhou Suncadia Biopharmaceuticals Co., Ltd, 10 μg/mL).

### Statistical analysis

Statistical analysis was performed using Medcalc V.15.0, SPSS V.26.0 (IBM) and GraphPad Prism V.8.0. Data were analysed with Student’s test, Mann–Whitney *U* test, Wilcoxon for two groups or ANOVA for multiple groups. For categorical variables, Chi-square test or Fisher’s exact test was applied. Kaplan–Meier method, Log-rank test and Cox regression analysis were used to determine the clinical significance. Data represented as means ± SD. A two-tailed *P* < 0.05 was considered as statistically significant.

## Results

### Dectin-1 is overexpressed and indicates poor prognosis in GC

**Fig. 1:**
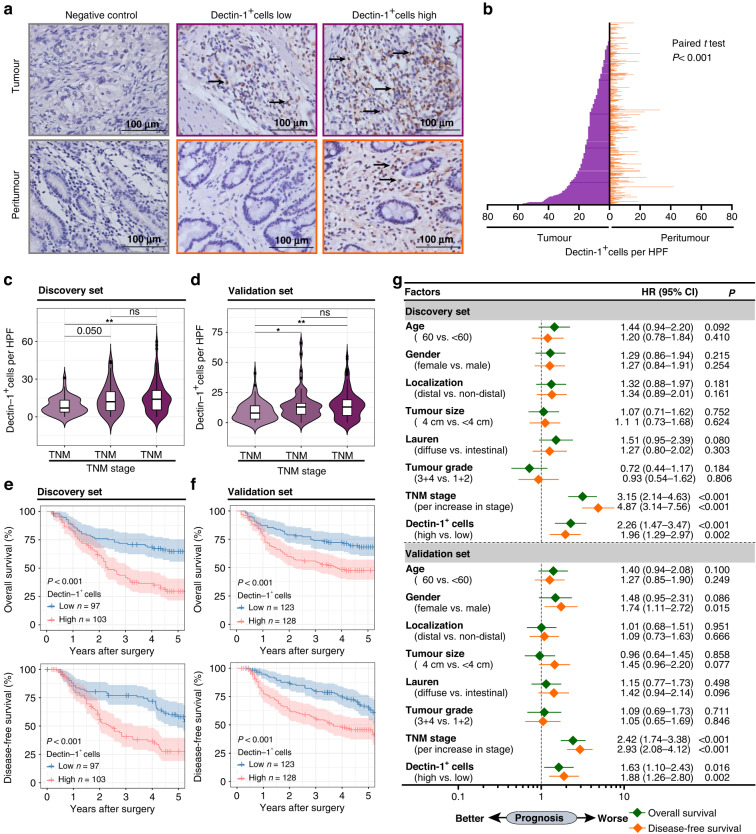
High expression of Dectin-1 predicts poor prognosis in GC. **a** Representative IHC staining images of Dectin-1^+^ cells in tumour tissues and peritumor tissues of GC (scale bar, 100 μm). **b** Quantification of Dectin-1^+^ cells in tumour tissues and corresponding peritumor tissues of GC (*n* = 451). Paired *t* test. **c**, **d** Quantification of Dectin-1^+^ cells in tumoral tissues among different stages of GC in Discovery set (*n* = 200) (**c**) and Validation set (*n* = 251) (**d**). One-way ANOVA, followed by Tukey’s multiple comparisons test. **e**, **f** OS and DFS of GC patients in Discovery set (**e**) and Validation set (**f**) based on the median score of Dectin-1^+^ cells. Log-rank test. **g** Multivariate analysis of Dectin-1^+^ cells and clinicopathological characteristics associated with OS and DFS in the Discovery set and Validation set. HR hazard ratio, CI confidence interval. *P < 0.05, ** *P* < 0.01, *** *P* < 0.001, ns refers to not significant.

We first evaluated the expression patterns of Dectin-1 in GC. TCGA-STAD and GEO datasets analysis indicated higher *CLEC7A* mRNA expression in tumour tissues compared with adjacent normal tissues (Supplementary Fig. S1A). Next, we detected Dectin-1 expression by IHC and found an increased presence of Dectin-1^+^ cells in tumour tissues, which verified the above findings (Fig. 1a, b). Furthermore, FCM results revealed that Dectin-1 was mainly expressed on CD45^+^ immune cells and was upregulated in tumour tissues compared to peritumor counterparts (Supplementary Fig. S1B, C).

Then, we sought to assess the clinical relevance of intratumoral Dectin-1^+^ cells in GC. FCM analysis demonstrated that the percentage of CD45^+^Dectin-1^+^ cells significantly increased as cancer progressed (Supplementary Fig. S1D). Similar observations were made when analysing the total number of Dectin-1^+^ cells in tumour tissues by IHC (Fig. 1c, d). We also analysed the infiltration of Dectin-1^+^ cells among different TNM stages in peritumor tissues, but no significant differences were observed (Supplementary Fig. S1E). We further investigated the prognostic value of Dectin-1^+^ cells in GC and found that patients with high infiltration of Dectin-1^+^ cells had significantly poorer OS and DFS than those with low infiltration of Dectin-1^+^ cells in both the Discovery set and Validation set (Fig. 1e, f). According to multivariate analysis, TNM stage and Dectin-1^+^ cells were identified as independent risk factors for OS and DFS in both the Discovery set and Validation set (Fig. 1g). Moreover, to compare the prognostic ability between TNM stage and Dectin-1^+^ cells, we applied the time-dependent area under the receiver operating characteristic (ROC) curve (AUC). Interestingly, the AUC was higher for TNM stage, and the prognostic ability of Dectin-1^+^ cells was inferior to TNM stage. Notably, the AUC of Dectin-1^+^ cells combined with the TNM stage was the highest in predicting either OS or DFS (Supplementary Fig. S1F, G). Collectively, these results suggest that Dectin-1 expression is upregulated and associated with disease progression in GC, and the infiltration of Dectin-1^+^ cells combined with the TNM stage can effectively improve the ability to predict the prognosis of GC patients.

### Dectin-1 is mainly expressed on TAMs in GC

**Fig. 2:**
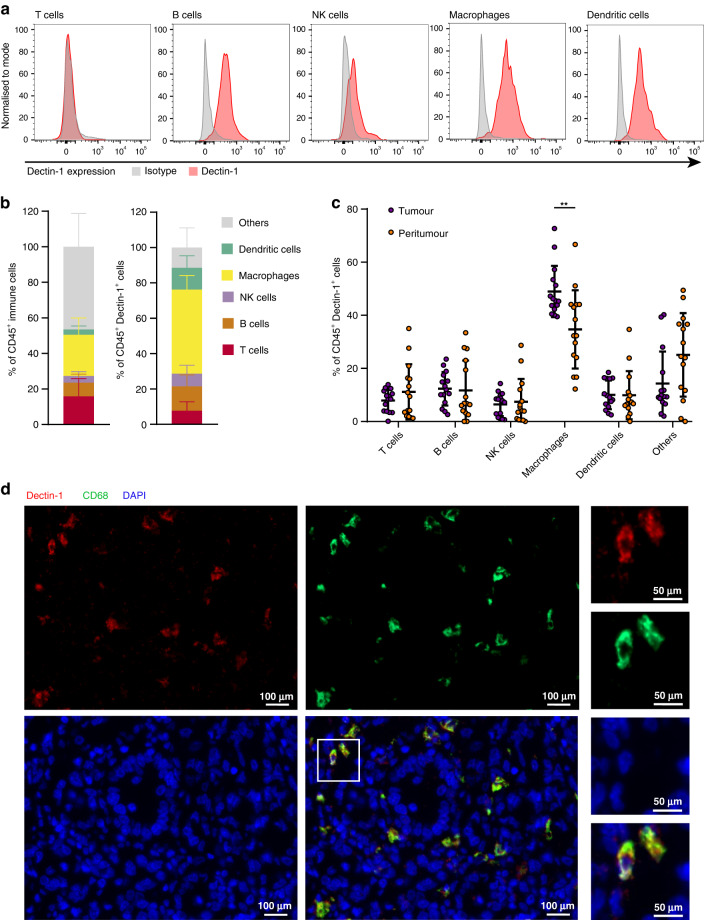
Dectin-1 is predominantly expressed on macrophages in GC. **a** Representative flow cytometric histograms showing the expression of Dectin-1 on gated tumour-infiltrating immune cell subsets in tumour tissues of GC. **b** Quantitative data on cellular composition of total CD45^+^ immune cells (left) and CD45^+^Dectin-1^+^ cells (right), including B cells (CD3^−^CD19^+^), T cells (CD3^+^CD19^−^), NK cells (CD3^−^CD56^+^), macrophages (CD68^+^) and DCs (HLA-DR^+^CD11c^+^) (*n* = 27). **c** The percentage of B cells, T cells, NK cells, macrophages and DCs among Dectin-1^+^ cells in tumour tissues and corresponding peritumor tissues of GC (*n* = 15). Paired *t* test. **d** Representative immunofluorescence staining of Dectin-1 (red), CD68 (green) and DAPI (blue) in tumour tissues of GC (scale bar, 100 μm). Dectin-1^+^ TAMs are circled with white frame (scale bar of the magnified image, 50 μm). **P < 0.01.

We next tried to assess the identity of Dectin-1^+^ cells in GC. FCM analysis revealed Dectin-1 was highly expressed on macrophages and DCs, but conservatively expressed on T, B and NK cells (Fig. 2a and Supplementary Fig. S2A). We further compared the composition of CD45^+^ immune cells and CD45^+^Dectin-1^+^ cells, and demonstrated that macrophages were the most important component of Dectin-1^+^ cells, accounting for more than 40% (Fig. 2b and Supplementary Fig. S2B, C). Notably, the percentage of macrophages among Dectin-1^+^ cells in tumour tissues was higher than that in peritumor tissues (Fig. 2c). However, we did not find significant differences in the percentage of T, B, NK cells and DCs (Fig. 2c). In addition, the co-expression of Dectin-1 with the TAM marker CD68 was further confirmed in FFPE GC tissues by immunofluorescence staining (Fig. 2d). Therefore, these data demonstrate that Dectin-1 is mainly expressed on TAMs in GC tissues.

### Dectin-1^+^ TAMs are associated with T-cell dysfunction in GC

**Fig. 3:**
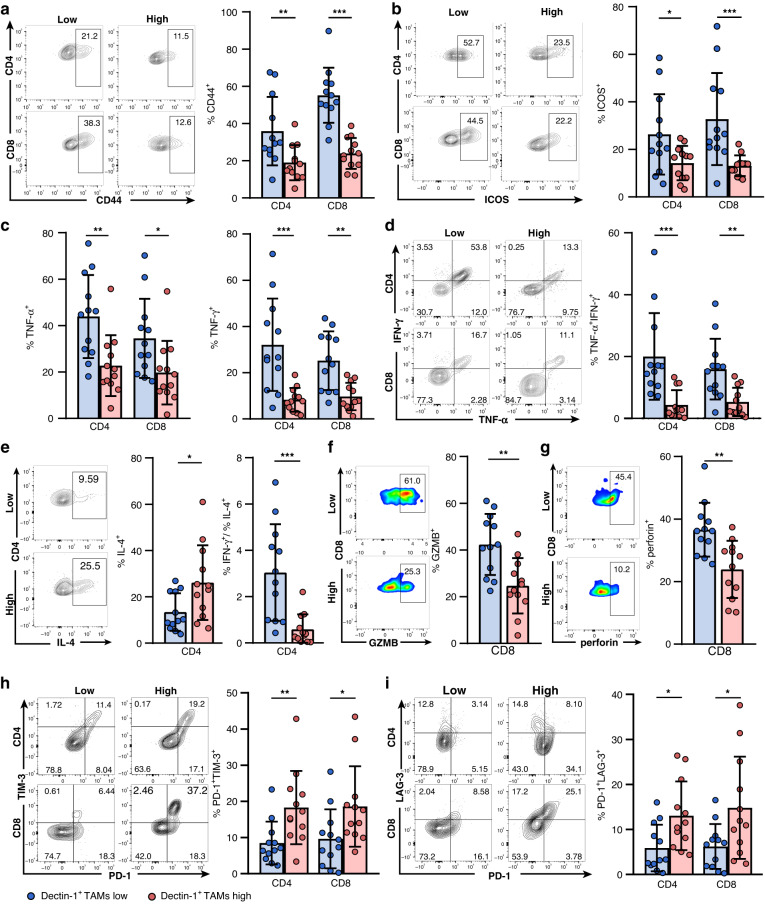
Dectin-1^+^ TAMs are positively corelated with impaired T-cell functions in GC. **a**, **b** The frequency of CD44^+^ (**a**) and ICOS^+^ (**b**) cells gated by CD4^+^ and CD8^+^ T cells in tumour tissues of GC (*n* = 12 per group). Representative flow cytometric plots (left) and quantitative data (right) are shown. **c** The frequency of TNF-α^+^ and IFN-γ^+^ cells gated by CD4^+^ and CD8^+^ T cells in tumour tissues of GC (*n* = 12 per group). **d** The frequency of TNF-α^+^IFN-γ^+^ cells gated by CD4^+^ and CD8^+^ T cells in tumour tissues of GC (*n* = 12 per group). Representative flow cytometric plots (left) and quantitative data (right) are shown. **e** The frequency of IL-4^+^ cells and the ratio of IFN-γ^+^ cells/IL-4^+^ cells gated by CD4^+^ T cells in tumour tissues of GC (*n* = 12 per group). Representative flow cytometric plots (left) and quantitative data (right) are shown. **f**, **g** The frequency of GZMB^+^ (**f**) and perforin^+^ (**g**) cells gated by CD8^+^ T cells in tumour tissues of GC (*n* = 12 per group). Representative flow cytometric plots (left) and quantitative data (right) are shown. **h**, **i** The frequency of PD-1^+^TIM-3^+^ (**h**) and PD-1^+^LAG-3^+^ (**i**) cells gated by CD4^+^ and CD8^+^ T cells in tumour tissues of GC (*n* = 12 per group). Representative flow cytometric plots (left) and quantitative data (right) are shown. Mann–Whitney *U* test. *P < 0.05, ** *P* < 0.01, *** *P* < 0.001, ns refers to not significant.

Based on the reported effects of TAMs on tumour-specific immunity [25], we attempted to investigate the relevance between Dectin-1^+^ TAMs and T-cell dysfunction. According to the median frequency of Dectin-1^+^ TAMs, GC patients were divided into Dectin-1^+^ TAMs low and high subgroup. We detected an increase in the percentage of CD4^+^ and CD8^+^ T cells in Dectin-1^+^ TAMs high subgroup than in Dectin-1^+^ TAMs low subgroup (Supplementary Fig. S3A), but the proliferation capacity remained unchanged (Supplementary Fig. S3B). Interestingly, despite the increased infiltration of T cells in Dectin-1^+^ TAMs high subgroup, their function was markedly attenuated. Specifically, compared with Dectin-1^+^ TAMs low subgroup, CD4^+^ and CD8^+^ T cells in Dectin-1^+^ TAMs high subgroup downregulated activation marker such as CD44 and inducible co-stimulator (ICOS) expression (Fig. 3a, b). In addition, the expression of CD4^+^ and CD8^+^ T-cell effector molecules, including tumour necrosis factor-α (TNF-α), interferon-γ (IFN-γ) and interleukin (IL)-2 was significantly reduced in Dectin-1^+^ TAMs high subgroup (Fig. 3c and Supplementary Fig. S3C). Notably, the multifunctional T-cell response in Dectin-1^+^ TAMs high subgroup was impaired, as measured by downregulating the co-expression of TNF-α and IFN-γ (Fig. 3d). We also found that CD4^+^ T cells in Dectin-1^+^ TAMs high subgroup exhibited elevated IL-4 expression, but not IL-10 or IL-17, with a Th2 phenotype and a lower Th1/Th2 ratio (IFN-γ^+^ cells/IL-4^+^ cells ratio in CD4^+^ T cells) (Fig. 3e and Supplementary Fig. S3D). In terms of CD8^+^ T cells, the cytolytic activity was significantly impaired in Dectin-1^+^ TAMs high subgroup, as evidenced by downregulated Granzyme B (GZMB) and perforin expression (Fig. 3f, g). Inhibitory receptor expression analysis revealed that CD4^+^ and CD8^+^ T cells in Dectin-1^+^ TAMs high subgroup upregulated the expression of PD-1, T-cell immunoglobulin and mucin domain 3 (TIM-3) and lymphocyte activation gene 3 (LAG-3) (Supplementary Fig. S3E–G). Furthermore, the percentages of exhausted T cells characterised by co-expression of PD-1 and TIM-3, or PD-1 and LAG-3 were also significantly increased (Fig. 3h, i) [26–28]. However, no significant differences in the expression of cytotoxic T-lymphocyte-associated antigen 4 (CTLA-4) and T-cell immunoreceptor with immunoglobulin and ITIM domain (TIGIT) were found between these two subgroups (Supplementary Fig. S3H, I). Taken together, these results suggest that Dectin-1^+^ TAMs were closely associated with T-cell immune tolerance in GC.

### Dectin-1^+^ TAMs display an immunosuppressive phenotype in GC

**Fig. 4:**
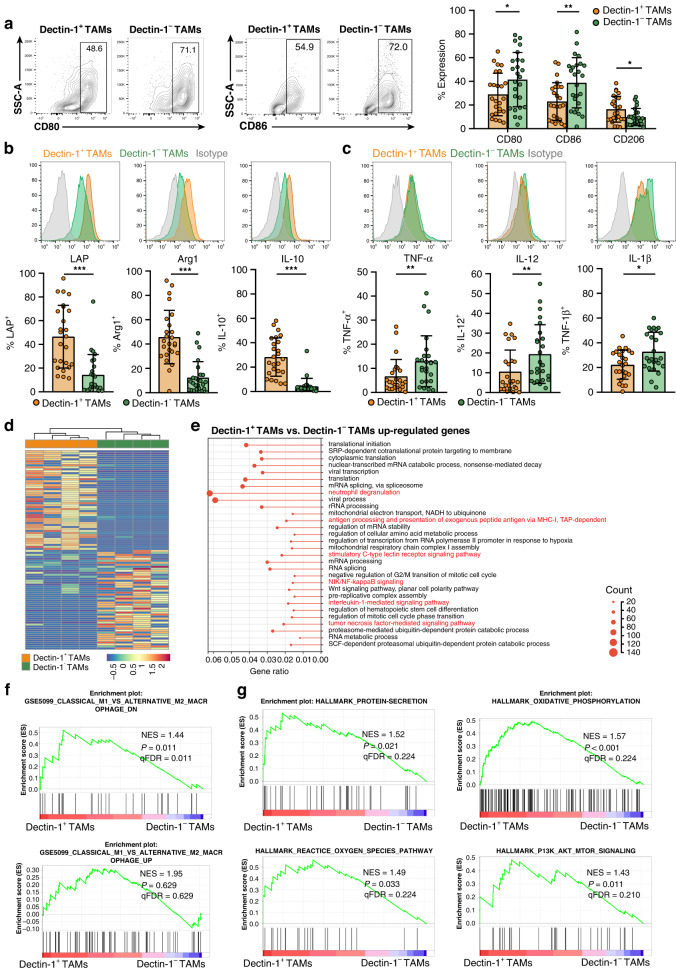
Dectin-1^+^ TAMs have an immunosuppressive phenotype. **a** The frequency of CD80^+^, CD86^+^ and CD206^+^ cells in Dectin-1^+^ TAMs versus Dectin-1^−^ TAMs in tumour tissues of GC (*n* = 25). Representative flow cytometric plots (left) and quantitative data (right) are shown. **b**, **c** The frequency of LAP^+^, Arg1^+^ and IL-10^+^ cells (**b**), as well as TNF-α^+^, IL-12^+^ and IL-1β^+^ cells (**c**) in Dectin-1^+^ TAMs versus Dectin-1^−^ TAMs in tumour tissues of GC (*n* = 25). Representative flow cytometric histograms (up) and quantitative data (down) are shown. Mann–Whitney *U* test. **d** Heatmap showing the top 100 DEGs between Dectin-1^+^ TAMs and Dectin-1^-^ TAMs (*n* = 4) in tumour tissues of GC. **e** GO analysis of upregulated DEGs between Dectin-1^+^ TAMs and Dectin-1^−^ TAMs associated with BP pathways. Red, enriched signalling pathways related to immune regulation. **f**, **g** GSEA of gene expression changes in Dectin-1^+^ TAMs versus Dectin-1^−^ TAMs. *P < 0.05, **P < 0.01, ***P < 0.001.

To further explore the mechanism of Dectin-1^+^ TAMs mediated immune evasion in GC, we characterised the phenotype of Dectin-1^+^ TAMs. FCM analysis revealed that Dectin-1^+^ TAMs exhibited relatively low expression of M1-like macrophage markers CD80 and CD86, while M2-like macrophage marker CD206 was highly expressed (Fig. 4a and Supplementary Fig. S4A). We also demonstrated elevated expression of immunosuppressive factors latency-associated peptide (LAP), arginase-1 (Arg1) and IL-10 in Dectin-1^+^ TAMs compared with Dectin-1^−^ TAMs (Fig. 4b). Conversely, Dectin-1^+^ TAMs downregulated pro-inflammatory cytokines, including TNF-α, IL-12 and IL-1β (Fig. 4c). Then, we employed RNA-seq analysis to assess the genome-wide gene expression features of purified Dectin-1^+^ TAMs and Dectin-1^−^ TAMs isolated from fresh GC tissues (Fig. 4d). GO analysis of BP pathways revealed that compared with Dectin-1^−^ TAMs, Dectin-1^+^ TAMs were enriched in immune signalling pathways, including neutrophil degranulation, antigen processing and presentation, stimulatory CLR signalling pathway, nuclear factor kappa B (NF-κB) signalling pathway, IL-1-mediated signalling pathway and TNF mediated signalling pathway (Fig. 4e). However, with the exception of the neutrophil degranulation pathway, no significant enrichments of immune signalling pathways were observed in Dectin-1^−^ TAMs (Supplementary Fig. S4B). This suggests that Dectin-1^+^ TAMs are closely related to immune regulation, whereas Dectin-1^−^ TAMs are heterogeneous. According to the results of GSEA, gene sets downregulated in M1-like macrophages compared to M2-like macrophages was enriched in Dectin-1^+^ TAMs (Fig. 4f). Furthermore, Dectin-1^+^ TAMs showed obviously upregulated gene sets involved in protein secretion, oxidative phosphorylation, reactive oxygen species (ROS) pathway and phosphatidylinositol-3-kinase (PI3K)-protein kinase B (AKT)-rapamycin target protein (mTOR) signalling pathways (Fig. 4g). Notably, both oxidative phosphorylation and the PI3K-AKT-mTOR signalling pathway are related to M2-like macrophage polarisation, whereas the ROS signalling pathway is associated with M1-like macrophage polarisation [29, 30]. Taken together, we concluded that despite the activation of several pro-inflammatory signalling pathways, Dectin-1^+^ TAMs were functionally proximal to M2-like macrophages.

### Dectin-1 blockade reactivates anti-tumour immunity in GC

**Fig. 5:**
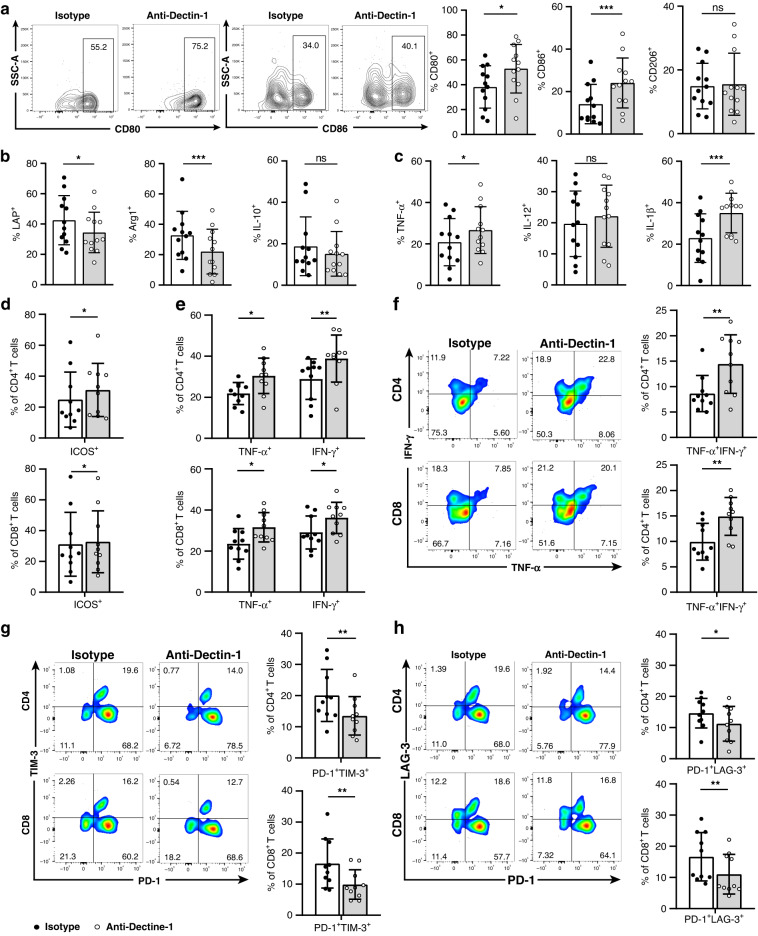
Dectin-1 blockade reactivates anti-tumour immunity in GC. Tumour single-cell suspensions were divided into Isotype group (Mouse IgG1 isotype control antibody, 10 μg/mL) and anti-Dectin-1 group (Human Dectin-1 monoclonal antibody, 10 μg/mL). Each treatment group was cultured in RPMI1640 medium (Gibco) containing 10% foetal bovine serum (Gibco) for 12 h at 37 °C. **a** The frequency of CD80^+^, CD86^+^, and CD206^+^ cells gated by Dectin-1^+^ TAMs after isotype and anti-Dectin-1 treatment in tumour tissues of GC (*n* = 12). Representative flow cytometric plots (left) and quantitative data (right) are shown. **b**, **c** The frequency of LAP^+^, Arg1^+^ and IL-10^+^ cells (**b**), as well as TNF-α^+^, IL-12^+^ and IL-1β^+^ cells (**c**) gated by Dectin-1^+^ TAMs after isotype and anti-Dectin-1 treatment in tumour tissues of GC (*n* = 12). **d**, **e** The frequency of ICOS^+^ (**d**) TNF-α^+^ and IFN-γ^+^ cells gated by CD4^+^ and CD8^+^ T cells (**e**) after isotype and anti-Dectin-1 treatment in tumour tissues of GC (*n* = 10). **f**–**h** The frequency of TNF-α^+^IFN-γ^+^ cells (**f**), PD-1^+^TIM-3^+^ (**g**) and PD-1^+^LAG-3^+^ (**h**) cells gated by CD4^+^ and CD8^+^ T cells after isotype and anti-Dectin-1 treatment in tumour tissues of GC (*n* = 10). Representative flow cytometric plots (left) and quantitative data (right) are shown. Wilcoxon test. *P < 0.05, ** *P* < 0.01, *** *P* < 0.001, ns refers to not significant.

After exploring the phenotype of Dectin-1^+^ TAMs, we wondered whether the blockade of Dectin-1 could influence the anti-tumour immunity in GC. To examine this, we evaluated the phenotype and function of Dectin-1^+^ TAMs and T cells in response to anti-Dectin-1 treatment. As shown in Fig. 5a, anti-Dectin-1 treatment induced increased expression of CD80 and CD86 on Dectin-1^+^ TAMs compared with the isotype control antibody treatment group, but the expression of CD206 remained unchanged. Intriguingly, anti-Dectin-1 treatment also promoted the secretion of pro-inflammatory cytokines TNF-α and IL-1β by Dectin-1^+^ TAMs, while attenuated the secretion of immunosuppressive factors LAP and Arg1 (Fig. 5b, c). To further investigate the impact of Dectin-1 blockade on T-cell activation, we examined the changes of T-cell status after anti-Dectin-1 treatment. The results showed that both CD4^+^ and CD8^+^ T cells were markedly activated, as evidenced by elevated expression of ICOS (Fig. 5d), but not CD44 (Supplementary Fig. S5A). Moreover, CD4^+^ and CD8^+^ T cells upregulated the expression of effector cytokines, including TNF-α, IFN-γ and IL-2 (Fig. 5e and Supplementary Fig. S5B). Meanwhile, the percentage of T cells co-expressing TNF-α and IFN-γ was also significantly increased after Dectin-1 blockade (Fig. 5f). We also found that anti-Dectin-1 treatment was able to downregulate the percentage of Th2 cells, reverse the Th1/Th2 ratio and increase the percentage of GZMB-expressing CD8^+^ T cells (Supplementary Fig. S5C–E). Conversely, the percentages of exhausted CD4^+^ and CD8^+^ T cells were significantly reduced after anti-Dectin-1 treatment (Fig. 5g, h). Taken together, these results demonstrate that blockade of Dectin-1 reprogrammes Dectin-1^+^ TAMs, as well as reactivates T cells in GC.

### Anti-PD-1 efficacy is enhanced in combination with Dectin-1 blockade

**Fig. 6:**
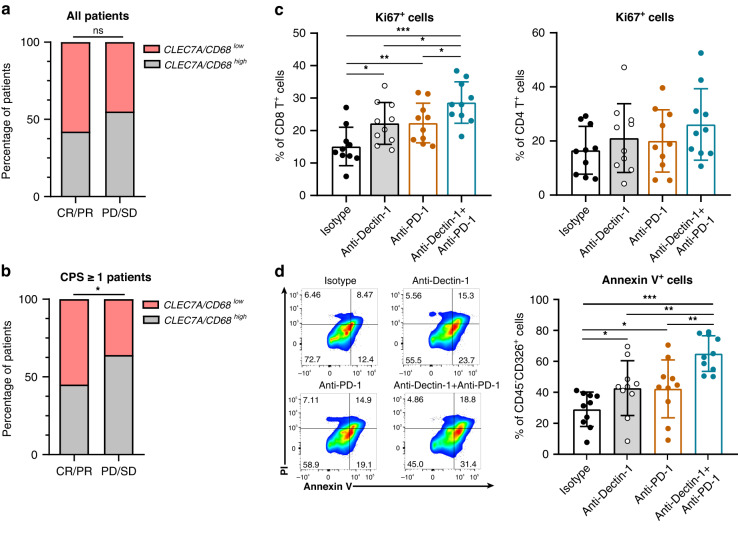
Dectin-1 blockade enhances anti-PD-1 efficacy. **a**, **b** Relationship between response to pembrolizumab and *CLEC7A/CD68* mRNA expression in all patients (*n* = 45) (**a**) and CPS ≥ 1 patients (*n* = 22) (**b**). Chi-square test. **c** The frequency of CD8^+^Ki67^+^ T cells (left) and CD4^+^Ki67^+^ T cells (right) after isotype, anti-Dectin-1, anti-PD-1, and anti-Dectin-1 + anti-PD-1 treatment in tumour tissues of GC (*n* = 10). **d** The frequency of apoptotic tumour cells after isotype, anti-Dectin-1, anti-PD-1, and anti-Dectin-1 + anti-PD-1 treatment in tumour tissues of GC (*n* = 10). Representative flow cytometric plots (left) and quantitative data (right) are shown. Mann–Whitney *U* test. *P < 0.05, **P < 0.01, ***P < 0.001, ns refers to not significant. CR complete response, PR partial response, PD progressive disease, SD stable disease.

Among the numerous immunotherapeutic strategies, PD-1/PD-L1 blockade has shown the most compelling clinical outcomes in GC [31]. Therefore, we next evaluated the impact of Dectin-1 blockade on response to anti-PD-1 therapy in GC. According to previous studies [32], we divided the amount of *CLEC7A* by the expression of *CD68* in each patient and formulated this value as the relative abundance of Dectin-1^+^ TAMs. Interestingly, we found that in PD-L1^+^ tumours (PD-L1 combined positive score (CPS) ≥1), patients belonging to *CLEC7A/CD68* high subgroup held more non-responders compared with *CLEC7A/CD68* low subgroup (Fig. 6a, b), indicating that Dectin-1^+^ TAMs might be involved in resistance to anti-PD-1 therapy. To test whether anti-Dectin-1 treatment could augment the therapeutic efficacy of PD-1/PD-L1 blockade, we next combined anti-Dectin-1 treatment with anti-PD-1 antibody. Tumour single-cell suspensions were treated with anti-Dectin-1 antibody either alone or in combination with anti-PD-1 antibody. We found that the combinatorial treatment of anti-Dectin-1 and anti-PD-1 resulted in a relatively larger increase in the proliferation ability of CD8^+^ T cells, while there was no significant change in CD4^+^ T cells (Fig. 6c). In addition, concurrent use of Dectin-1 and PD-1 blockade promoted tumour cell apoptosis more than either monotherapy or isotype treatment (Fig. 6d). Overall, these results suggest the possibility of administering anti-Dectin-1 as an immunotherapeutic adjuvant to overcome the resistance to ICIs.

## Discussion

Numerous studies highlighted the generation, accumulation, and activation of TAMs in the regulation of tumour immunology and immunotherapy [33]. TAMs have been reported to prime T cells and generate anti-tumour immunity in vivo. In addition, tumorigenic TAMs are considered as potent immunosuppressors to inhibit T-cell-mediated anti-tumour immune responses through diverse mechanisms within the tumour microenvironment. In this study, we discovered that Dectin-1 was highly expressed in GC tumour tissues, and the abundance of Dectin-1^+^ cells was associated with disease progression and poor prognosis of GC patients. Further analysis revealed that Dectin-1^+^ cells were mainly composed of TAMs, and high levels of Dectin-1^+^ TAMs were associated with T-cell immune tolerance. In addition, we confirmed that blocking Dectin-1 could reprogramme Dectin-1^+^ TAMs and reactivate the anti-tumour immune response of T cells. These results indicate that Dectin-1-expressing TAMs play an important role in GC immunotherapy and are expected to be potential targets.

The mechanisms of how Dectin-1 affect TAMs in GC remain unclear. Here, we demonstrated that Dectin-1^+^ TAMs in GC were responsible for expressing more LAP, Arg1 and IL-10, while secreting less TNF-α, IL-12 and IL-1β compared with Dectin-1^−^ TAMs. Our previous study showed that LAP was able to regulate the secretion and activation of TGF-β and was involved in the construction of T-cell dysfunction in GC microenvironment [34]. Therefore, LAP expressed by Dectin-1^+^T AMs might play an important role in mediating immune evasion of GC. Dectin-1^+^ TAMs also have been shown to express Arg1, which metabolises L-arginine required for activation of T cells to urea and L-ornithine, thereby inhibiting T-cell proliferation and activation [35]. Moreover, the mitogen and stress-activated protein kinases (MSKs) in macrophages were activated by zymosan and promoted Dectin-1-mediated IL-10 production, which was controlled by the ability of MSKs to phosphorylate the transcription factor CREB [36]. As a potent anti-inflammatory factor, IL-10 plays an important role in negatively regulating Th1/Th2 balance and effector function of CD8^+^ T cells [37, 38]. Consistent with studies that reduced Th1/Th2 ratio might indicate poor prognosis in GC patients [39], we demonstrated that the high infiltration of Dectin-1^+^ TAMs was associated with Th1/Th2 imbalance. In addition, Dectin-1 regulates the production of more complex cytokines when it collaborates with other pattern recognition receptors or interacts with different ligands [40]. Zymosan can induce immune tolerance by stimulating macrophages to secrete TGF-β in a toll-like receptor 2 (TLR2) and Dectin-1-dependent manner [41]. Furthermore, specific tumour-associated glucans have been described as potential endogenous Dectin-1 ligands. Chiba et al. showed Dectin-1 binds to N-glycan structures on the surface of tumour cells, that contributes to the eventual cytolytic activation of NK cells [42]. In pancreatic cancer, Dectin-1^+^ TAMs can regulate the expression of Arg1 by binding to galectin-9 [20]. Notably, glycosylation alterations are frequent during the development and progression of GC [43]. Thus, it is possible that blockade of the Dectin-1 signalling pathway or its interaction with specific ligand could affect certain macrophage subsets to regulate immune response in GC. Future biochemical experiments will be important to characterise the specific mechanisms of Dectin-1^+^ TAMs that mediate immune evasion in GC.

Immunotherapy using anti-PD-1/PD-L1 antibodies has been shown to unleash T-cell effector functions to control GC progression. However, the response rate in patients with advanced GC is still not satisfactory [44]. Given the profound impact of TAMs on immunotherapy, numerous therapeutic strategies targeting for TAMs have been developed to complement checkpoint blockade [45]. The strategies are broadly divided into two categories: reducing the number of TAMs and repolarizing TAMs [25]. Paradoxically, complete depletion of TAMs with colony-stimulating factor-1 receptor (CSF-1R) inhibitors may result in the loss the immunostimulatory properties of phagocytosis and antigen presentation, and therefore cannot yield therapeutic benefits as monotherapy [25]. In comparison, TAM reprogramming has shown more promising results for optimising immunotherapy regimens. For example, blocking the CD47-SIRPα axis using targeted monoclonal antibodies restores macrophage-mediated phagocytosis [46]. Treatment with TLR agonists also results in reprogramming of TAMs and triggers potent inflammatory responses, which may not only protect the host against infections, but may also promote anti-tumour immunity [47]. Dectin-1 is the first example of a non-TLR pattern recognition receptor that can mediate its own intracellular signals [48]. Studies have shown that Dectin-1 expression enhances TLR-mediated activation of NF-κB by β-glucan-containing particles in macrophages and DCs, mediating synergistic production of cytokines such as IL-12 and TNF-α [49]. Dectin-1 also triggers production of ROS, an inflammatory response that is primed by TLR activation [50]. Additionally, other study has demonstrated that dectin-1 activation robustly dampens TLR-induced pro-inflammatory signature in macrophages [51]. Despite the success of these strategies, their efficacy in clinical trials remains limited [7].To provide novel insights into reprograming TAMs compensate for ICIs, we demonstrated that blocking Dectin-1 could reverse the immunosuppressive effects of Dectin-1^+^ TAMs and restore T-cell effector function using an established in vitro model of the GC microenvironment [52].Of great intrigue, our study suggested that Dectin-1^+^ TAMs might be involved in resistance to anti-PD-1 therapy. Notably, we found the gene expression features of Dectin-1^+^ TAMs are also involved in the regulation of PI3K-AKT-mTOR signalling. Recent study has shown that the number of mutated genes in the PI3K-AKT-mTOR pathway ≥2 was identified as a potential predictor of primary resistance to ICIs in the mismatch repair-deficient (dMMR)/microsatellite instability-high (MSI-H) gastric adenocarcinomas [53]. Therefore, the PI3K-AKT-mTOR pathway hyperactivated in Dectin-1^+^ TAMs may functionally induce resistance to anti-PD-1 immunotherapy in GC. Importantly, we demonstrated the combinatorial treatment with anti-PD-1 and anti-Dectin-1 antibody elicit a synergistic effect in promoting CD8^+^ T-cell proliferation and tumour cell apoptosis, suggesting that Dectin-1^+^ TAMs were expected to be immunotherapeutic targets and enhanced the anti-tumour effects of ICIs. Here, we proposed that Dectin-1 blockade might provide a new therapeutic approach that broadens the scope for TAM targeting in tumours. Further studies on the association of Dectin-1^+^ TAMs and immunotherapy resistance are warranted in the future.

In conclusion, we demonstrate that Dectin-1 can be used as an independent prognostic indicator in GC and is mainly expressed on TAMs. Mechanically, Dectin-1^+^ TAMs upregulated expression of multiple anti-inflammatory cytokines, which might account for T-cell tolerance in GC. These findings provide new approaches to develop therapies targeting Dectin-1.

## Supplementary information

Supplementary Materials

## Data Availability

Data and materials generated that are relevant to the results are included in this article. Other data are available from the corresponding author Prof. Xu upon reasonable request.
