# Supplementary material for: C-type lectin receptor Dectin-1 blockade on tumour-associated macrophages improves anti-PD-1 efficacy in gastric cancer
Source: Br J Cancer. 2023 Jul 8;129(4):721–32. doi: 10.1038/s41416-023-02336-5 (PMC10421860; doi:10.1038/s41416-023-02336-5)
Supplement: Supplementary file 1 — Supplementary Materials [file 41416_2023_2336_MOESM1_ESM.doc]

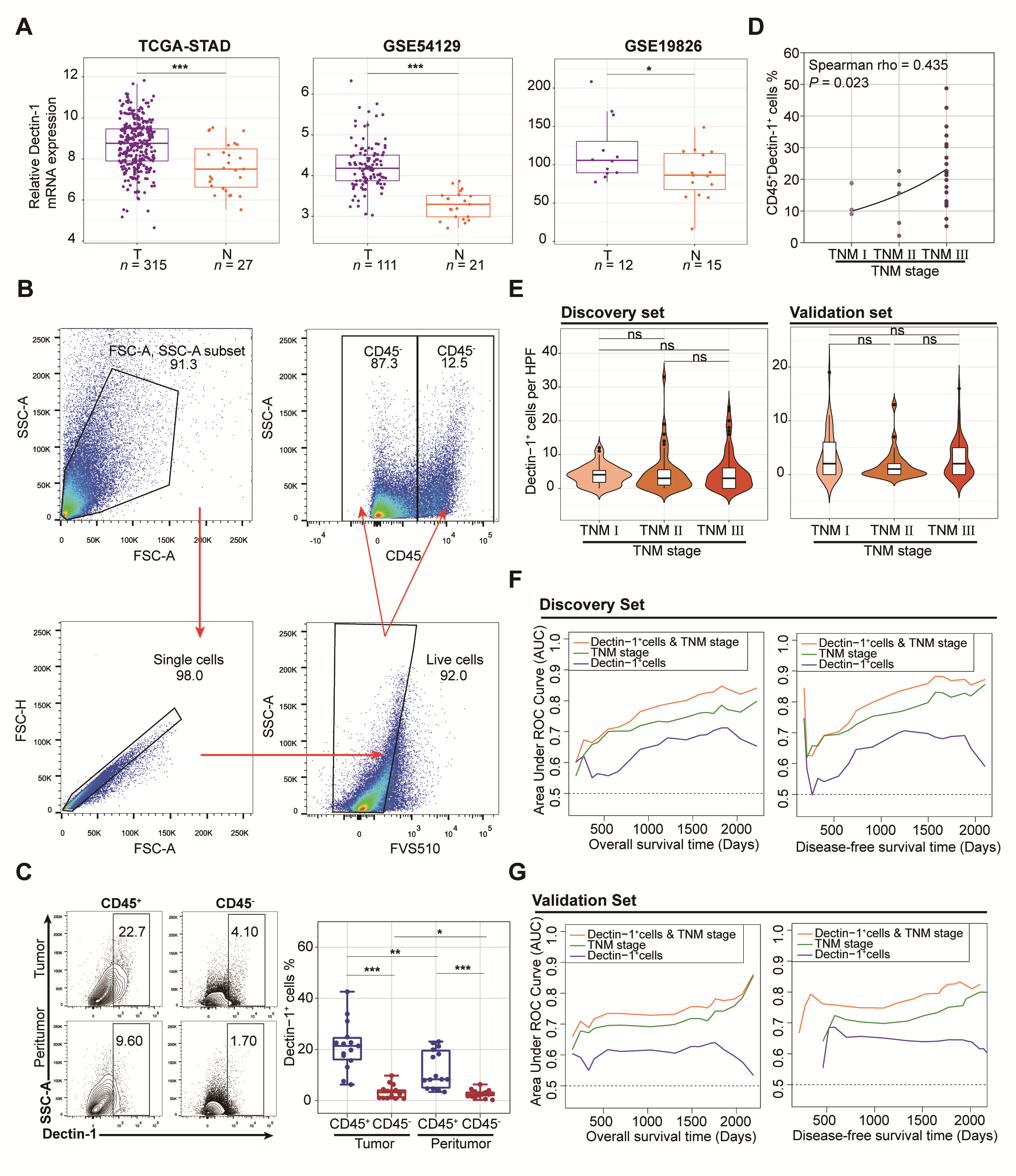


**Supplementary Figure 1. Expression patterns of Dectin-1 in GC.** **(A)** *CLEC7A* mRNA expression in TCGA-STAD and GEO datasets in tumor tissues and normal tissues of GC. Un-paired *t* test. **(B)** Gating strategy for CD45^+^ cells and CD45^-^ cells. **(C)** The frequency of Dectin-1^+^ cells among CD45^+^ immune cells and CD45^-^ non-immune cells in tumor tissues and corresponding peritumor tissues of GC. One-way ANOVA, followed by Tukey’s multiple comparisons test (*n* = 15). Representative flow cytometric plots (left) and quantitative data (right) were shown. **(D)** The frequency of CD45^+^Dectin-1^+^ cells in different stages of GC (*n* = 27). Correlation coefficients were tested by Spearman’s rho. **(E)** Quantification of Dectin-1^+^ cells in peritumor tissues among different stages of GC in Discovery set (*n* = 200) and Validation set (*n* = 251). **(F-G)** Time-dependent area under the receiver operating characteristic (ROC) curve (AUC) was conducted to investigate the predictive power of TNM stage, Dectin-1^+^ cells, and Dectin-1^+^ cells combined with TNM stage on OS and DFS in Discovery set **(F)** and Validation set **(G).** **P* < 0.05, ** *P* < 0.01, *** *P* < 0.001, ns refers to not significant.


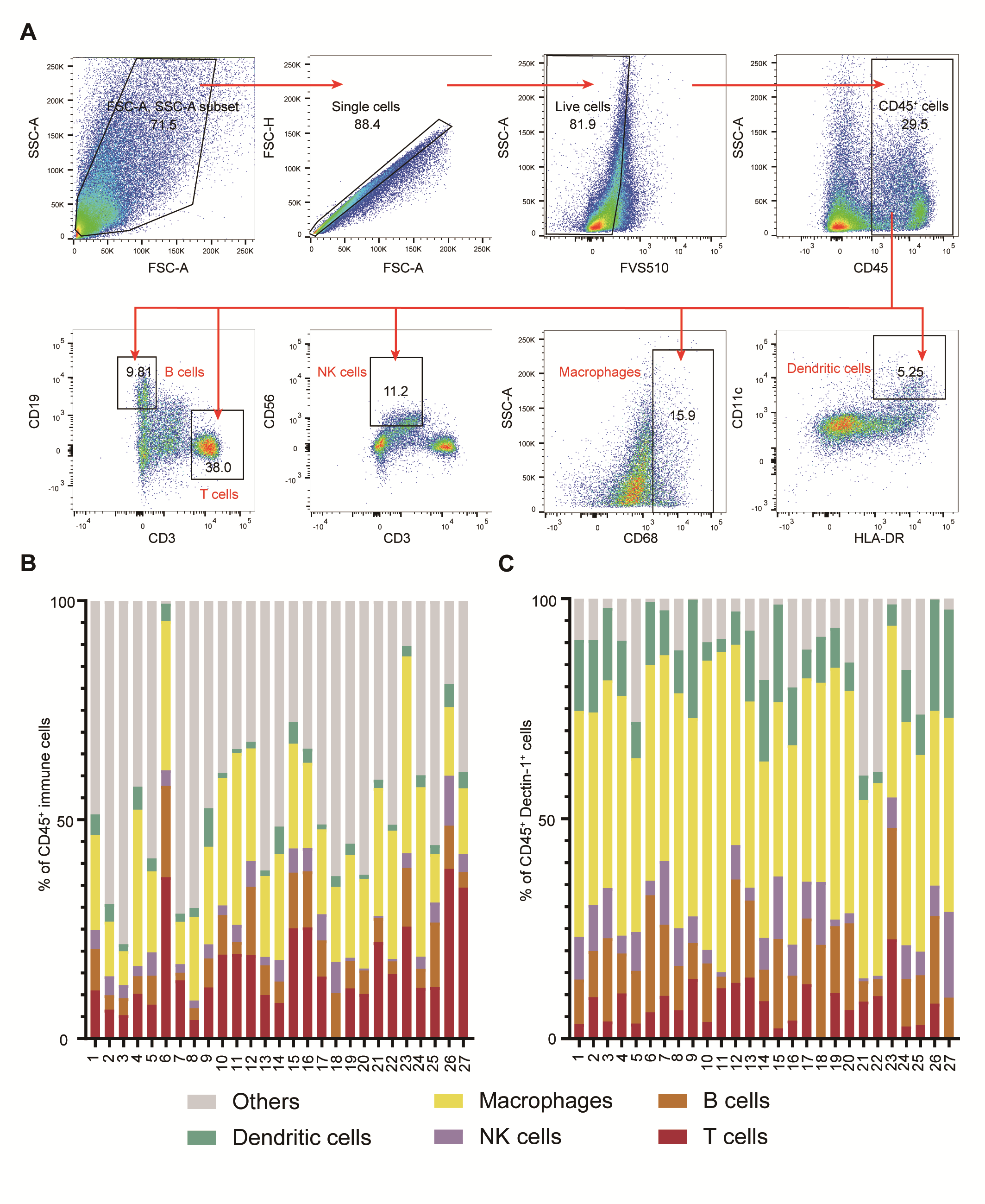


**Supplementary Figure 2. Cell composition of CD45^+^ immune cells and CD45^+^Dectin-1^+^ cells. (A)** Gating strategy and representative flow cytometric plots of cell composition of CD45^+^ immune cells in tumor tissues of GC, including B cells (CD3^-^CD19^+^), T cells (CD3^+^CD19^-^), NK cells (CD3^-^CD56^+^), macrophages (CD68^+^) and dendritic cells (DCs, HLA-DR^+^CD11c^+^). **(B-C)** The immune cell subpopulations of CD45^+^ immune cells **(B)** and CD45^+^Dectin-1^+^ cells **(C)** in tumor tissues of GC, including B cells, T cells, NK cells, macrophages and DCs. Results are shown as the percentage of immune cell subpopulations (*n* =27). Colors reflect distinct immune cell subpopulations as determined by indicated cell markers.


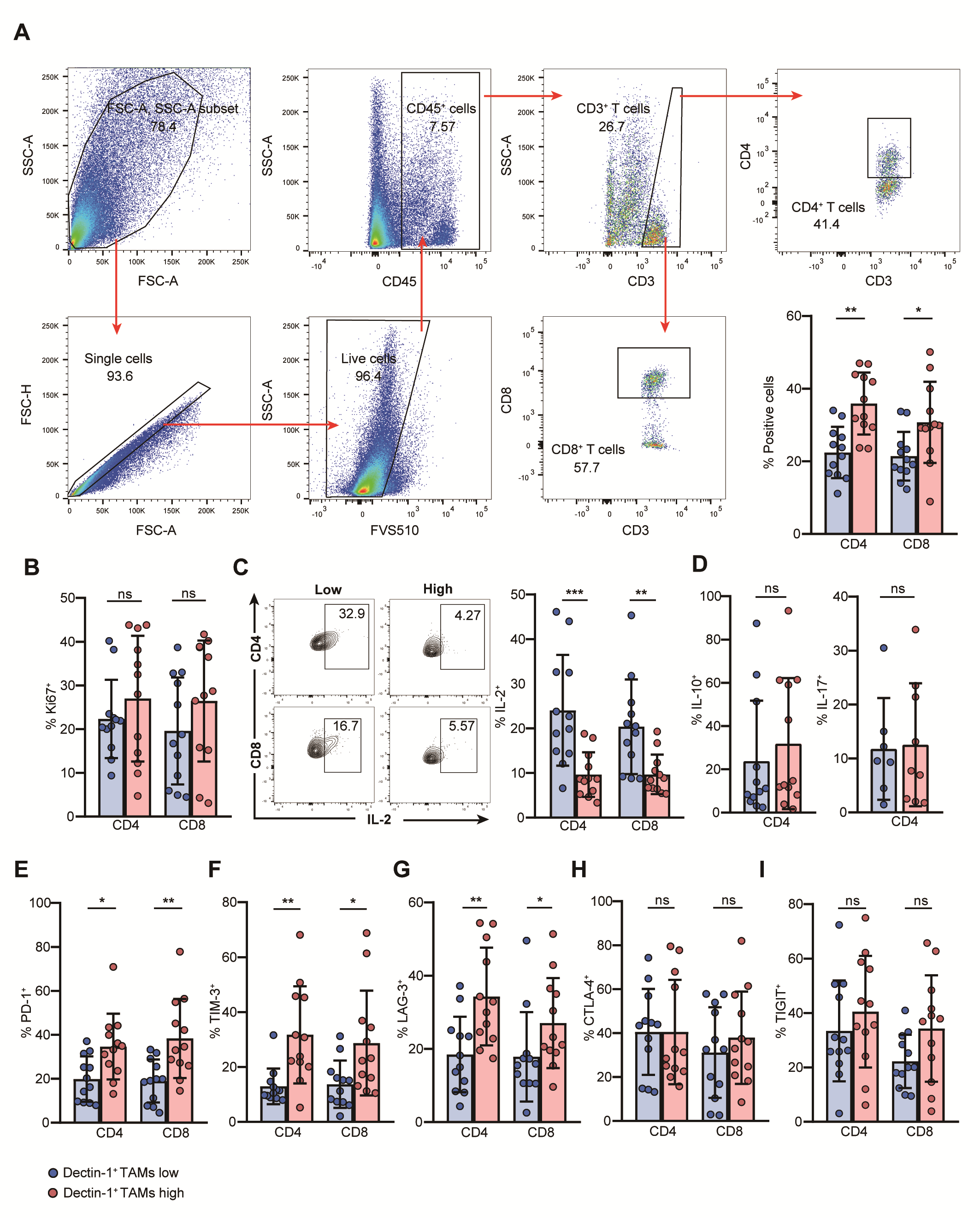


**Supplementary Figure 3. Dectin-1^+^ TAMs are associated with T cell dysfunction in GC. (A)** Gating strategy for CD4^+^ and CD8^+^ T cells. The frequency of CD4^+^ and CD8^+^ T cells in tumor tissues of GC (*n* = 12 per group). **(B)** The frequency of Ki67^+^ cells gated by CD4^+^ and CD8^+^ T cells in tumor tissues of GC (*n* = 12 per group). **(C)** The frequency of IL-2^+^ cells gated by CD4^+^ and CD8^+^ T cells in tumor tissues of GC (*n* = 12 per group). Representative flow cytometric plots (left) and quantitative data (right) were shown. **(D)** The frequency of IL-10^+^ and IL-17^+^ cells was detected by gated CD4^+^ T cells in tumor tissues of GC (*n* = 12 per group). **(E-I)** The frequency of PD-1^+^ **(E)**, TIM-3^+^ **(F)**, LAG-3^+^ **(G)**, CTLA-4^+^ **(H)** and TIGIT^+^ **(I)** cells was detected by gated CD4^+^ and CD8^+^ T cells in tumor tissues of GC (*n* = 12 per group). Mann-Whitney U test. **P* < 0.05, ***P* < 0.01, ****P* < 0.001, ns refers to not significant.


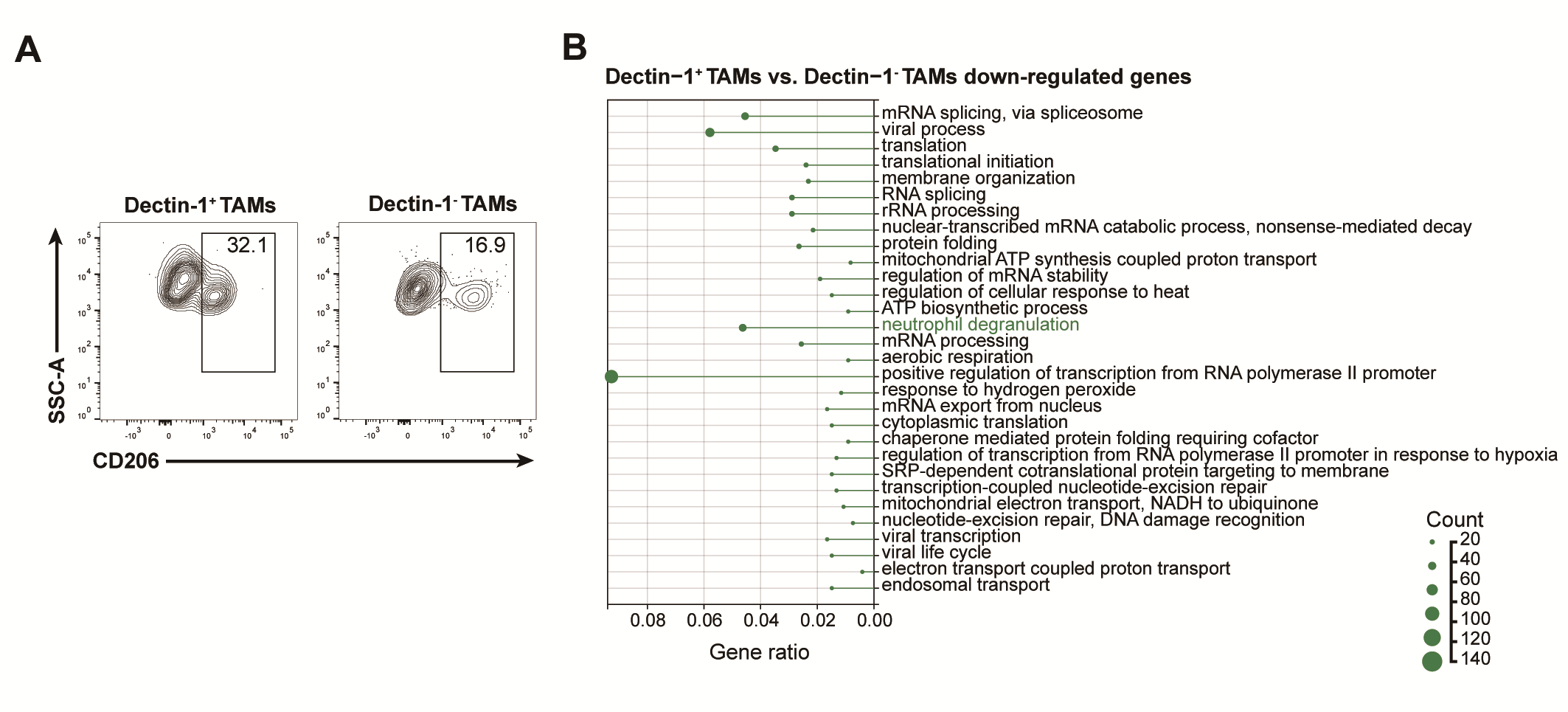


**Supplementary Figure 4. Differences in signaling pathways between Dectin-1^+^ TAMs and Dectin-1^-^ TAMs. (A)** Representative flow cytometric plots of CD206^+^ cells in Dectin-1^+^ TAMs versus Dectin-1^-^ TAMs in tumor tissues of GC. **(B)** Gene Ontology analysis of downregulated DEGs between Dectin-1^+^ TAMs and Dectin-1^-^ TAMs associated with BP. Green, enriched signaling pathways related to immune regulation.


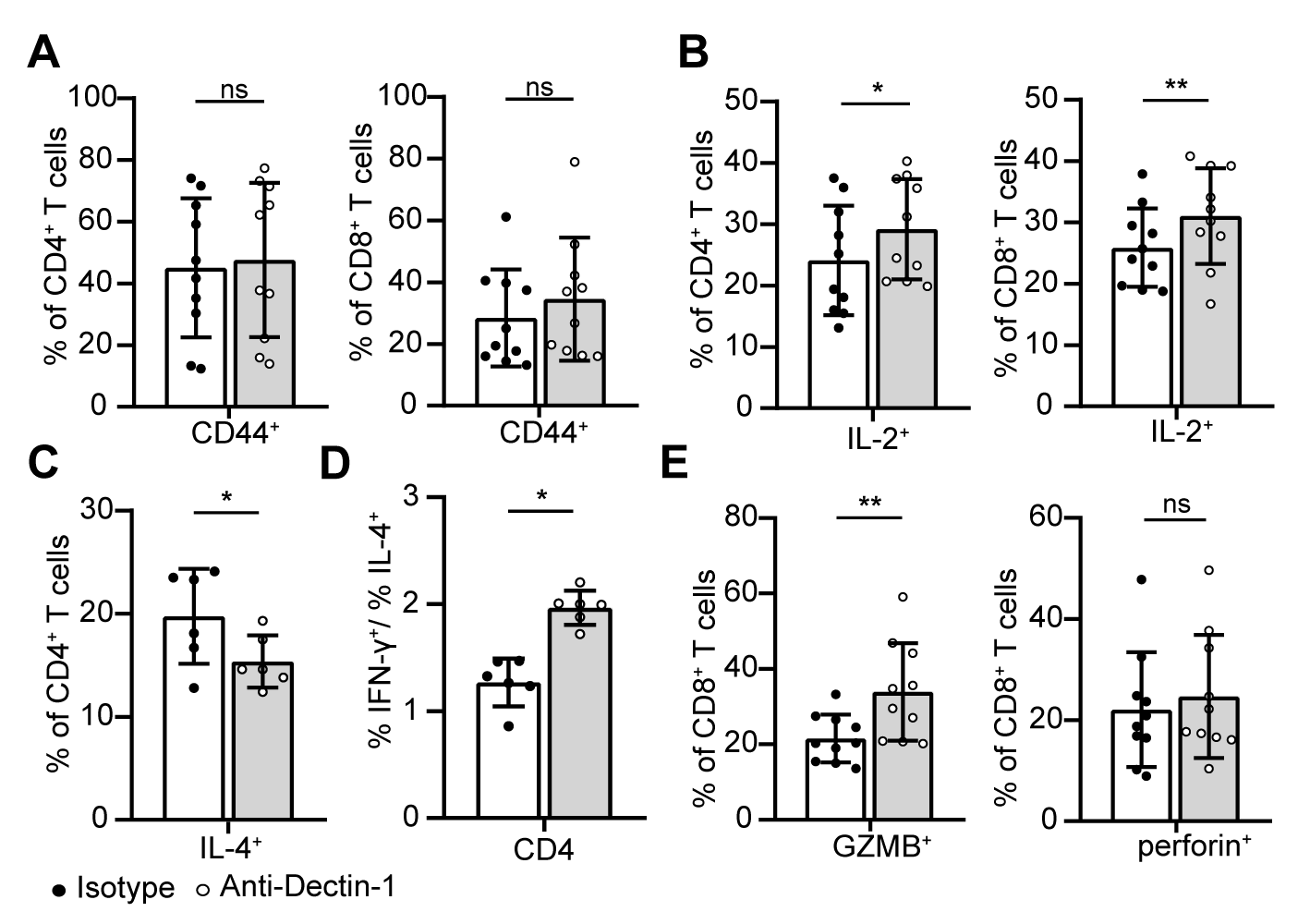


**Supplementary Figure 5. Dectin-1 blockade reactivates anti-tumor immunity in GC. (A-B)** The frequency of CD44^+^ **(A)** and IL-2^+^ cells **(B)** gated by CD4^+^ and CD8^+^ T cells after isotype and anti-Dectin-1 treatment in tumor tissues of GC (*n* = 10). **(C-D)** The frequency of IL-4^+^ cells **(C)** and the ratio of IFN-γ^+^ cells/IL-4^+^ cells **(D)** gated by CD4^+^ T cells after isotype and anti-Dectin-1 treatment in tumor tissues of GC (*n* = 6). **(E)** The frequency of GZMB^+^ and perforin^+^ cells gated by CD8^+^ T cells after isotype and anti-Dectin-1 treatment in tumor tissues of GC (*n* = 10). Wilcoxon test. * *P* < 0.05, ** *P* < 0.01.

**Supplementary Table 1. Associations between Dectin-1^+^ cells and clinicopathological characteristics in patients with GC.**

| **Factors** | **Discovery set (*n* = 200)** | | | **Validation set (*n* = 251)** | | |
| --- | --- | --- | --- | --- | --- | --- |
|  | **Low (*n* = 97)** | **High (*n* = 103)** | ***P*** | **Low (*n* = 123)** | **High (*n* = 128)** | ***P*** |
| Age at surgery (year)^a^ |  |  | 0.883 |  |  | 0.193 |
| Median (IQR) | 59 (53-69) | 58 (50-70) |  | 63 (54-71) | 59 (53-68) |  |
| Gender |  |  | 0.750 |  |  | 0.744 |
| Male | 61 | 67 |  | 92 | 98 |  |
| Female | 36 | 36 |  | 31 | 30 |  |
| Localization |  |  | 0.719 |  |  | 0.776 |
| Non-distal | 39 | 44 |  | 44 | 48 |  |
| Distal | 58 | 59 |  | 79 | 80 |  |
| Tumor size (cm)^a^ |  |  | 0.859 |  |  | 0.948 |
| Median (IQR) | 3.5 (2.5-5.0) | 4.0 (2.5-5.0) |  | 3.0 (2.0-5.0) | 3.0 (2.0-4.5) |  |
| Lauren’s classification |  |  | 0.745 |  |  | 0.681 |
| Intestinal type | 60 | 66 |  | 79 | 79 |  |
| Diffuse type | 37 | 37 |  | 44 | 49 |  |
| Grade |  |  | 0.368 |  |  | 0.708 |
| G1 | 6 | 3 |  | 9 | 5 |  |
| G2 | 22 | 19 |  | 24 | 26 |  |
| G3 | 69 | 81 |  | 89 | 96 |  |
| G4 | 0 | 0 |  | 1 | 1 |  |
| pT stage |  |  | **0.003** |  |  | **0.004** |
| T1 | 21 | 12 |  | 34 | 20 |  |
| T2 | 20 | 8 |  | 23 | 12 |  |
| T3 | 18 | 19 |  | 22 | 26 |  |
| T4 | 38 | 64 |  | 44 | 70 |  |
| pN stage |  |  | 0.211 |  |  | 0.393 |
| N0 | 40 | 35 |  | 55 | 45 |  |
| N1 | 10 | 6 |  | 16 | 18 |  |
| N2 | 21 | 21 |  | 21 | 22 |  |
| N3 | 26 | 41 |  | 31 | 43 |  |
| TNM stage |  |  | **0.009** |  |  | **0.005** |
| Ⅰ | 29 | 15 |  | 45 | 24 |  |
| Ⅱ | 26 | 23 |  | 27 | 30 |  |
| Ⅲ | 42 | 65 |  | 51 | 74 |  |
| Adjuvant chemotherapy |  |  | 0.175 |  |  | 0.359 |
| No | 43 | 36 |  | 58 | 53 |  |
| Yes | 54 | 67 |  | 65 | 75 |  |

^a^Modeled as a continuous variable.

**Supplementary Table 2. Antibodies used for flow cytometry and fluorescence-activated cell sorting analysis.**

| **Antibodies** | **Clone** | **Source** | **Catalog No.** |
| --- | --- | --- | --- |
| APC/Cy7 anti-human CD45 Antibody | 2D1 | Biolegend | 368516 |
| PerCP/Cy5.5 anti-human CD369 Antibody | 15E2 | Biolegend | 355408 |
| APC anti-human CD68 Antibody | Y1/82A | Biolegend | 333810 |
| PerCP/Cy5.5 anti-human CD3 Antibody | HIT3a | Biolegend | 300328 |
| PE/Cy7 anti-human CD19 Antibody | HIB19 | Biolegend | 302216 |
| PE anti-human CD56 Antibody | 5.1H11 | Biolegend | 362508 |
| BV785 anti-human HLA-DR Antibody | L243 | Biolegend | 307642 |
| BV605 anti-human CD11c Antibody | 3.9 | Biolegend | 301636 |
| BV605 anti-human CD4 Antibody | SK3 | Biolegend | 344646 |
| FITC anti-human CD8 Antibody | RPA-T8 | BD | 555366 |
| AF700 anti-human CD8 Antibody | SK1 | Biolegend | 344724 |
| PE/Cy7 anti-human Ki67 Antibody | B56 | BD | 561283 |
| PE anti-human CD44 Antibody | BJ18 | Biolegend | 338808 |
| APC anti-human ICOS Antibody | C398.4A | Biolegend | 313510 |
| BV421 anti-human TNF-α Antibody | MAb11 | Biolegend | 502932 |
| BV786 anti-human IFN-γ Antibody | 4S.B3 | BD | 563731 |
| PE/Cy7 anti-human IL-2 Antibody | MQ1-17H12 | Biolegend | 500326 |
| APC anti-human IL-4 Antibody | 8D4-8 | Biolegend | 500714 |
| AF700 anti-human IL-17A Antibody | BL168 | Biolegend | 512318 |
| AF647 anti-human GZMB Antibody | GB11 | Biolegend | 515406 |
| PE anti-human Perforin Antibody | δG9 | BD | 556437 |
| AF700 anti-human Perforin Antibody | B-D48 | Biolegend | 353324 |
| PE anti-human PD-1 Antibody | A17188B | Biolegend | 621608 |
| BV650 anti-human TIM-3 Antibody | 7D3 | BD | 565564 |
| BV785 anti-human LAG-3 Antibody | 11C3C65 | Biolegend | 369322 |
| PE/Cy7 anti-human CTLA-4 Antibody | BNI3 | Biolegend | 369614 |
| BV421 anti-human TIGIT Antibody | A15153G | Biolegend | 372710 |
| PE/Cy7 anti-human CD80 Antibody | 2D10 | Biolegend | 305218 |
| BV650 anti-human CD86 Antibody | IT2.2 | Biolegend | 305428 |
| FITC anti-human CD206 Antibody | 19.2 | BD | 551135 |
| PE/Cy7 anti-human LAP (TGF-β1) Antibody | TW4-2F8 | Biolegend | 349610 |
| PE anti-human ARG1 Antibody | 14D2C43 | Biolegend | 369704 |
| BV650 anti-human IL-10 Antibody | JES3-9D7 | BD | 564051 |
| FITC anti-human IL-12 Antibody | 27537 | eBioscience | MA5-23683 |
| PE anti-human IL-1β Antibody | CRM56 | eBioscience | 12-7018-82 |
| APC anti-human CD326 (EpCAM) Antibody | 9C4 | Biolegend | 324208 |
| FITC anti-human CD14 Antibody | M5E2 | Biolegend | 301804 |
| PE anti-human CD369 Antibody | 15E2 | Biolegend | 355404 |
